# Phosphatidlyinositol-3-kinase C2 beta (PI3KC2β) is a potential new target to treat IgE mediated disease

**DOI:** 10.1371/journal.pone.0183474

**Published:** 2017-08-18

**Authors:** Shekhar Srivastava, Zhai Li, Edward Y. Skolnik

**Affiliations:** 1 Division of Nephrology, New York University Langone Medical Center, New York, New York, United States of America; 2 Department of Molecular Pathogenesis, Skirball Institute for Biomolecular Medicine, New York University Langone Medical Center, New York, New York, United States of America; 3 The Helen L. and Martin S. Kimmel Center for Biology and Medicine, Skirball Institute for Biomolecular Medicine, New York University Langone Medical Center, New York, New York, United States of America; 4 Department of Pharmacology, Skirball Institute for Biomolecular Medicine, New York University Langone Medical Center, New York, New York, United States of America; University of Torino, ITALY

## Abstract

Cross linking of the IgE receptor (FcεRI) on mast cells plays a critical role in IgE-dependent allergy including allergic rhinitis, asthma, anaphylaxis, and delayed type hypersensitivity reactions. The Ca^2+^ activated K^+^ channel, KCa3.1, plays a critical role in IgE-stimulated Ca^2+^ entry and degranulation in mast cells by helping to maintain a negative membrane potential, which provides an electrochemical gradient to drive Ca^2+^ influx. Of the 3 classes of PI3K, the class II PI3Ks are the least studied and little is known about the roles for class II PI3Ks *in vivo* in the context of the whole organism under normal and pathological conditions. Studying bone marrow derived mast cells (BMMC) isolated from *PI3KC2β*^*-/-*^ mice, we now show that the class II PI3KC2β is critical for FcεRI stimulated KCa3.1 channel activation and the subsequent activation of mast cells. We found FcεRI-stimulated Ca^2+^ entry, cytokine production, and degranulation are decreased in BMMC isolated from *PI3KC2β*^*-/-*^ mice. In addition, *PI3KC2β*^*-/-*^ mice are markedly resistant to both passive cutaneous and passive systemic anaphylaxis. These findings identify PI3KC2β as a new pharmacologic target to treat IgE-mediated disease.

## Introduction

Mast cells play important roles in a variety of immune and inflammatory reactions, which include immediate-type hypersensitivity reactions, allergy, asthma, autoimmunity, and resistance to infection[1, 2]. Upon stimulation of the high affinity IgE (FcεRI) receptor, mast cells rapidly degranulate and release a diverse array of chemicals and compounds, which include histamine, proteoglycans, and prostaglandins[2],[3]. Mast cells also release a number of cytokines such as IL-4 and IL-13, which promote Th2 development and TNF and IL-1, which promote the local inflammatory response. As a result, new pharmacologic targets that inhibit FcεRI activation of mast cells are likely to have therapeutic potential in IgE mediated and other diseases[3, 4].

Influx of extracellular Ca^2+^ following FcεRI activation is critical for mast cell activation, which is mediated by FcεRI stimulated IP3-mediated depletion of endoplasmic reticulum Ca^2+^ stores leading to activation of calcium release activated release (CRAC) channels and Ca^2+^ influx[5]. One of the consequences of Ca^2+^ influx is depolarization of the membrane, which if left unchecked, limits further Ca^2+^ influx by removing the favorable electrochemical gradient to drive further Ca^2+^ influx. Thus, mast cells require the K^+^ channels KCa3.1 which, by effluxing K^+^, maintains a hyperpolarized membrane potential critical for sustaining the gradient for Ca^2+^ entry via CRAC channels[5].

We previously identified a signaling pathway from the FcεRI and the T cell receptor to activation of KCa3.1. This pathway includes activation of the class II PI3K, PI3KC2β, resulting in the generation of PI3P, which is required for NDPK-B to phosphorylate KCa3.1 at histidine 358 leading to its activation[6–9]. In addition, we found that the E3 ubiquitin ligase, TRIM27, negatively regulates mast cell activation by ubiquitinating and inhibiting PI3K-C2β[10]. Of the 3 classes of PI3K, the class II PI3Ks are the least studied and characterized and consist of 3 isoforms PI3KC2α, PI3KC2β and PI3KC2γ[11, 12]. While numerous studies have indicated that each isoform has distinct functions, there have been only a limited number of studies of the 3 isoforms in knockout animals and as a result little is known about the roles for class II PI3Ks under normal and disease conditions *in vivo* in the context of the whole organism[12]. Unlike the other two class II PI3Ks, *PI3KC2α*^*-/-*^ mice are embryonically lethal indicating an essential role in development[13], while studies of selective knockouts of PI3KC2*α* have identified roles in angiogenesis[13], primary cilia formation[14], dysregulated platelets adehsions[15], and vascular integrity[13]. *PI3KC2γ*^*-/-*^ mice exhibit reduced liver accumulation of glycogen and develop insulin resistance, hyperlipidemia and adiposity on a high fat diet, which is at least partly due to impaired activation of AKT2[16]. Studies of *PI3KC2β*^*-/-*^ mice have unexpectedly demonstrated that while *PI3K-C2β*^*-/-*^ mice exhibit no overt phenotype[17], they exhibit increased insulin sensitivity and glucose tolerance, and are resistant to high fat diet induced steatosis[18]. These surprising and divergent roles for PI3K-C2γ and PI3K-C2β in insulin signaling and response to a high fat diet highlight essential roles for these kinases in regulating critical biological processes and underscores the importance in studying class II PI3K knockouts under both physiological and stressed conditions in the context of various disease models.

To address whether pharmacological inhibition of PI3K-C2β may be a new target to treat IgE mediated diseases by impairing activation of mast cells, we undertook studies in *PI3K-C2β*^*-/-*^ mice. These studies confirmed genetically that PI3K-C2β plays a critical role in FcεRI activation of mast cells by mediating the activation of KCa3.1 thereby facilitating Ca^2+^ influx and the subsequent activation of bone marrow derived mast cells (BMMC). Moreover, *PI3K-C2β*^*-/-*^ mice were resistant to IgE mediated passive systemic and cutaneous anaphylaxis. Taken together, these findings reinforce the potential for inhibitors of PI3K-C2β to be possible new treatment for allergy and other IgE mediated diseases.

## Materials and methods

### Cells and constructs

Bone marrow derived mast cells (BMMC) were generated from 6–8 week old *PI3KC2β*^*-/-*^ and *PI3KC2β*^*+/+*^ mice that were backcrossed 8 generations to C57Bl/6 mice[17]. Exons 3–5 were deleted in *PI3KC2β*^*-/-*^ mice which led to a non-functional truncated protein lacking the kinase and C2 domains. Genotyping was performed by PCR on genomic DNA with specific primers for the deleted allele (P1, 5′-TGTTAGAACCTGCCGCCTTTAC-3’, and P2, 5′-CCGAATCAGCCTCATTTCCTCTC-3′) and for the wild-type allele (P3, 5′-GGCACACACTAACCACAGCACC-3′, and P4, 5′-TCGATGCACGT CTCTCC GC-3′). The PCR product for the deleted allele is 404 bp, whereas the product for the wild-type allele is 251 bp. Bone marrow cells were cultured for 6–8 weeks in RPMI supplemented with IL-3 (20 ng/ml), stem cell factor (100 ng/ml), and 10% FCS[7]. Generation of a pure population of mast cells after 6 weeks of culture were verified by staining with PE-labeled anti-FcεRI antibody followed by FACS analysis.

### Whole cell patch clamp

Whole cell patch clamping was performed on PI3KC2β^+/+^ and PI3KC2β^-/-^ BMMCs that were first sensitized overnight with anti-DNP IgE and then stimulated with DNP-HSA using conditions previously described[19]. Briefly the standard pipette solution contained 140 mM KCl, 2 mM MgCl_2_, 10 mM Hepes, 2 mM Na^+^-ATP and 0.1 mM GTP, pH 7.3. The standard external solution contained 140 mM NaCl, 5 mM KCl, 2 mM CaCl_2_, 1 mM MgCl_2_ and 10 mM Hepes, pH 7.3. Whole-cell currents were recorded using an Axoclamp 200 B amplifier (Axon Instruments, Foster City, CA, USA), and currents were evoked by applying voltage commands to a range of potentials in 10 mV steps from a holding potential of –20 mV.

To verify that PI3KC2β mediated activation of KCa3.1 via the generation of PI3P, PI3P (100 nM) was added into the pipette solution during patch clamping of PI3KC2β^-/-^ BMMCs. PI(3)P diC16 [C_41_H_45_Na_3_O_16_P_2_] or diC4 C_17_H_29_Na_3_O_16_P_2_], and PI(3,4)P_2_ [C_41_H_76_Na_5_O_19_P_3_] were purchased from Echelon Biosciences and used according to manufactures specifications at a concentration of 100 nM in the pipette solution. The lipids were resuspended in water and flash frozen in liquid nitrogen and stored at -20°C between the uses.

### Intracellular Ca^2+^ activity

BMMCs from *PI3KC2β*^*-/-*^ and *PI3KC2β*^*+/+*^ mice were sensitized overnight with anti-DNP IgE (100 ng/ml) and subsequently loaded with 5 μM Fura-2 AM ester (Molecular Probes) in RPMI medium for 30 min at room temperature, washed and then resuspended in RPMI. Cells were attached to poly(L)lysine-coated coverslips for 20 min in a RC-20 bath flow chamber (Warner Instrument Corp., Hamden, CT) and fura-2 fluorescence was recorded (Delta Ram; PTI Inc., South Brunswick, NJ) at excitation wavelengths of 340 and 380 nm. Data are represented as the normalized 340/380 ratio after background subtraction. Intracellular Ca^2+^ was measured before and after the perfusion of DNP-HSA in the HBSS buffer in the presence of 1 mM extracellular Ca^2+^.

### Membrane potential

To measure membrane potential amphotericin (240 μg/ml) was added in the pipette solution (same as described in whole cell patch clamp) and current clamp mode was used [20]. Perforation was monitored by the increase in the ability to compensate for cell capacitance and by the decrease in the series resistance. BMMCs from *PI3KC2β*^*-/-*^ and *PI3KC2β*^*+/+*^ mice were sensitized overnight with anti-DNP IgE (100 ng/ml) and after measuring the basal membrane potential, DNP-HSA 1μg/ml was used to measure the changes in the membrane potential.

### β-hexosaminidase release and cytokine production

BMMCs were plated at 1 X 10^6^ cells/96 well plate in media supplemented with 1 μg/ml DNP-IgE antibody and kept at 37^0^ C in the incubator overnight. Cells were then washed and stimulated with various concentrations of DNP-HSA for 30 minutes in Tyrode’s buffer (10 mM HEPES, pH 7.4, 130 mM NaCl, 5 mM KCl, 1.4 mM CaCl_2_, 1 mM MgCl_2_, 5.6 mM glucose and 0.1% (wt/vol) BSA. Cells were spun at 1200 RPMs and β-hexosaminadase was measured in the supernatant by incubating 30 μl of supernatant with 3.3 μl of p-nitrophenyl-N-acetyl-β-D-glucosamide (10 mM) diluted in 0.2 M citrate buffer, pH 4.5 for 1.5 hours at 37^0^ C. The reaction was then stopped by adding 135 μl of a 0.1 M Na_2_CO_3_/0.1 M NaHCO_3_ solution and then assayed on an ELISA plate reader at an OD @ 405 nm. β-hexosaminadase was measured in the pellet following a similar protocol with the exception that the cell pellet was lysed in Tyrode’s buffer with 0.1% triton.

To assay for cyokines, mast cells were stimulated as above, total RNA was isolated using Trizol reagent and then reverse transcribed using random hexamer primers. Quantitative PCR was then assessed using SYBR Green 1 by iCycler iQ (Biorad) using cytokine specific primers purchased from Qiagen. Cytokines were also measured in the supernatants obtained before and 24 hrs after the stimulation of the cells using the ELISA ready-set-go kit from ebioscience for different cytokines. Cytokines were measured according to the manufacturer’s protocol.

### Passive systemic and cutaneous anaphylaxis

To assess whether *PI3KC2β*^*-/-*^ mice are resistant to passive systemic anaphylaxis, *PI3KC2β*^*+/+*^ and *PI3KC2β*^*-/-*^ mice were first sensitized with anti-DNP IgE (1μg/g body weight) administered by intraperitoneal injection. After 5 hrs, mice were challenged with either DNP-HSA (100 μg) or PBS control and body temperature was measured before and then at 7 minute intervals following challenge using a rectal probe[21]. Blood was also collected 30 minutes following challenge and assayed for histamine as described[22].

To assess passive cutaneous anaphylaxis, mice were sensitized intradermally with anti-DNP IgE and 24 hours later were injected intravenously with DNP-HSA containing 0.5% Evan’s blue dye. 30 minutes after dye injection, mice were sacrificed and tissue sections around the intradermal injection site were excised and weighed. Evan’s blue dye was then extracted from the tissue by incubation of biopsies in 0.5 ml formamide at 55°C for 24 h and quantitated by absorbance at 620 nm[23].

All mice were housed in the NYU School of Medicine Central Animal Facility. Mice are maintained in accordance with the Animal Welfare Act, the United States Department of Agriculture Regulations (9 CFR, Parts 1, 2, and 3), and the Guide for the Care and Use of Laboratory Animals (National Academy Press, Revised 1996). New York University School of Medicine has a currently approved Animal Welfare Assurance Agreement (No. A3435-01) with the NIH Office for Protection from Research Risks. NYUSM has been awarded Full Accreditation by AAALAC International (2/27/2001). To ensure there was no discomfort, animals were anesthetized with isoflurane (3–5%) during the experiments. All animals were followed daily for any signs of infection or distress such as respiratory distress, non-motility, failure to groom, hunched appearance and, if so, animals were euthanized based on these humane indications by CO_2_ gas using a cage chamber, followed by cervical dislocation, consistent with the AVMA Panel on Euthanasia.

All experiments were approved by the Office of Science and Research Institutional Animal Care and Use Committee at New York University School of Medicine under Laboratory Animal Protocol 140106–02.

## Results

### FcεRI activation of KCa3.1 and Ca^2+^ influx in *PI3KC2β*^*-/-*^ mice

**Fig 1:**
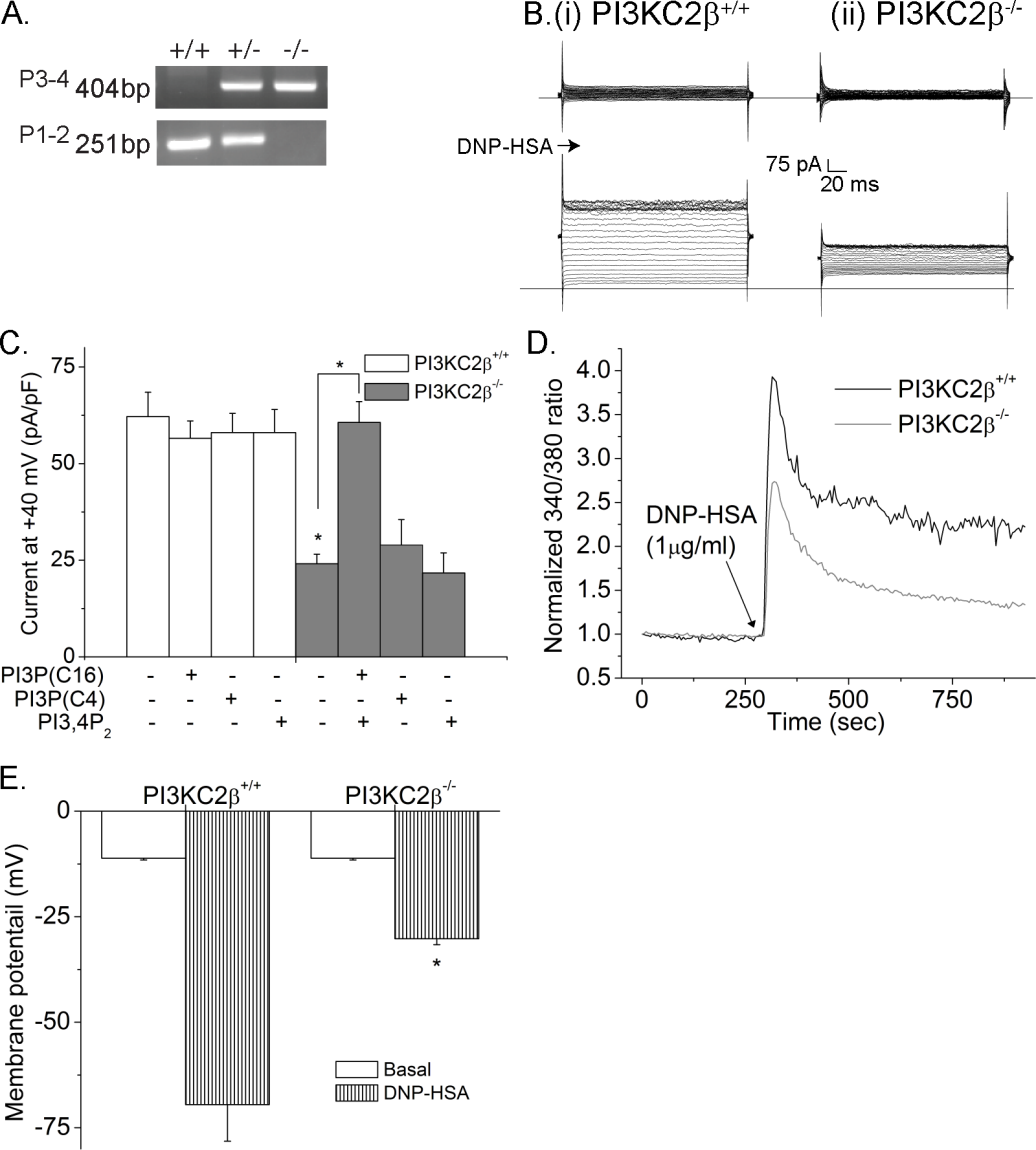
FcεRI activation KCa3.1 channel activation and Ca^2+^ influx is impaired in BMMC derived from *PI3KC2β*^*-/-*^ mice. A Genotyping of from *PI3KC2β*^*+/+*^ and *PI3KC2β*^*-/-*^ mice (see Material and Methods). The amplicon with primer pair 1 and 2 is 251 bp for the wild-type allele. The amplicon with primer pair 3 and 4 is 404 bp for the null allele. B. BMMC from *PI3KC2β*^*+/+*^ and *PI3KC2β*^*-/-*^ mice were sensitized with anti-DNP IgE, and whole cell patch clamp was performed with or without stimulation of FcεRI with DNP-HSA. C. Bar graph summary of whole-cell patch-clamp experiments performed in B at +40 mV. n = 12 cells each. To verify that the decrease in KCa3.1 channel activity in PI3KC2β^-/-^ BMMCs was due to the decreased levels of PI3P, rescue of channel activity was assessed after addition of 100 nM of PI3P (C16 or C4) or PI3,4P_2_ to the pipette solution during patch clamping. All experiments shown are representative of at least three experiments performed on cells isolated from three separate mice. *p<0.05 as compared to *PI3KC2β*^*+/+*^ mice or as indicated. Mast cells were loaded with Fura-2 AM (5 mM) and Ca^2+^ flux was determined after cross-linking with DNP-HSA as described in B.

*PI3K-C2β*^*-/-*^ mice have been previously described[17] and were generated by crossing *PI3K-C2β* ^*fl/fl*^ mice with *EIIa-cre* mice. *PI3K-C2β* ^*fl/fl*^ mice lack exons 3–5 which includes the PIK, kinase, and C2 domains and would be predicted to encode a truncated unstable protein **(Fig 1A**). *PI3K-C2β*^*-/-*^ mice were phenotypically normal and born at the expected Mendelian ratio. Bone marrow derived mast cells (BMMC) were isolated from *PI3K-C2β*^*-/-*^ mice that had been back crossed 10 generations with C57Bl/6 mice. FcεRI stimulated KCa3.1 activation (**Fig 1B and 1C**) and Ca^2+^ influx (**Fig 1D**) were decreased in PI3K-C2β^-/-^ BMMC when compared with PI3K-C2β^+/+^ BMMC. Consistent with the decreased KCa3.1 current and calcium flux in PI3K-C2β^-/-^ BMMC, these cells were also significantly depolarized following FcεRI stimulation (**Fig 1E**). The decrease in KCa3.1 current was due to decreased PI3P because channel activity could be rescued by dialyzing PI3P (C16), but not PI3P (C4) or PI3,4P_2_ (**Fig 1C**), into PI3KC2β^-/-^ BMMC during whole cell patch clamp. Rescue of KCa3.1 current by PI3P required insertion of PI3P into membranes; Dipalmitoyl PI3P (C16) which contains a 16 carbon acyl group and inserts into membranes rescued while water soluble dibutanoyl PI3P(C4) which contains a 4 carbon acyl group and does not insert into membranes did not (**Fig 1C**).

### FcεRI stimulated β-hexosaminidase release and cytokine production is decreased in PI3KC2β^-/-^ BMMCs

**Fig 2:**
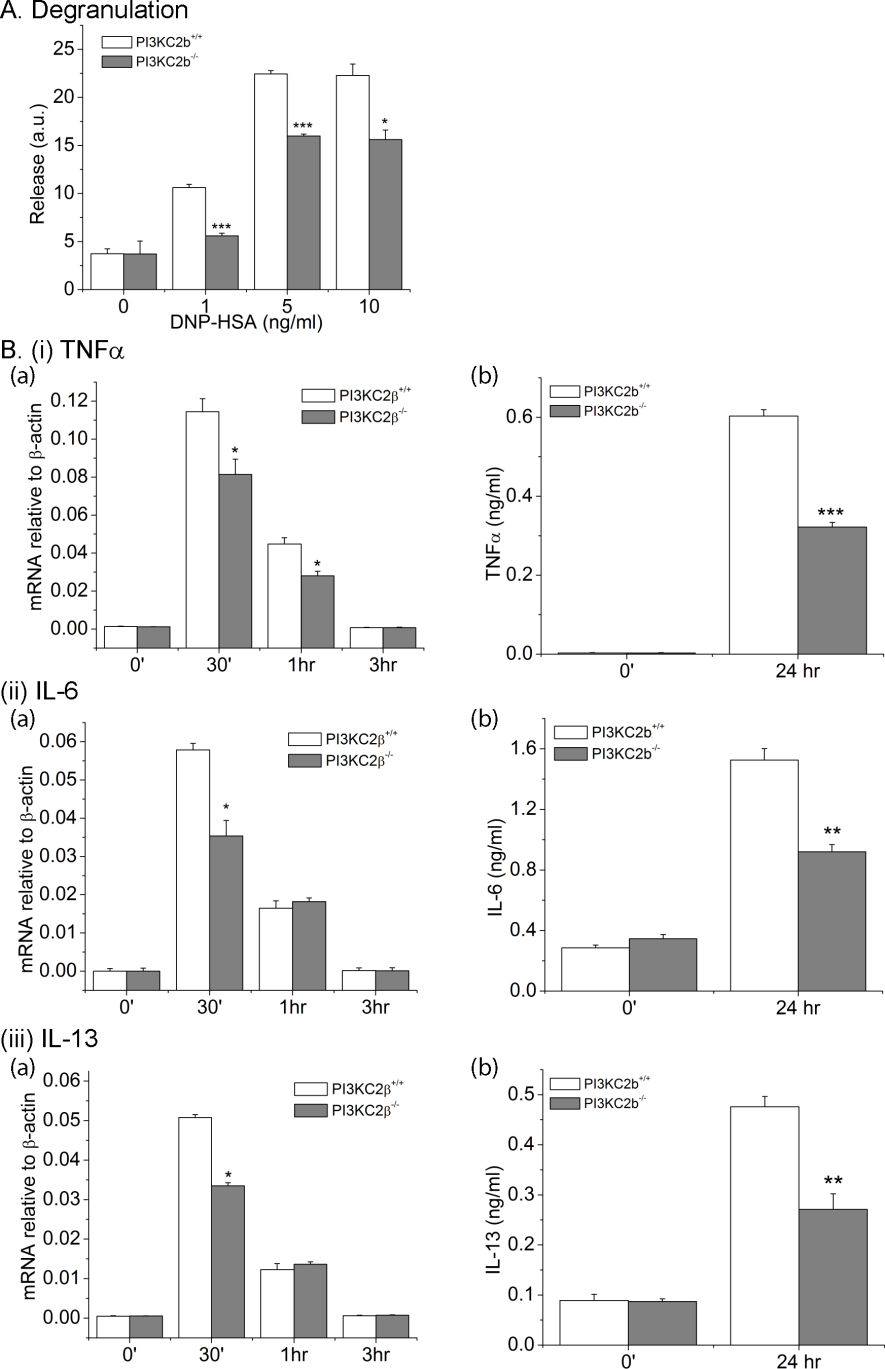
FcεRI stimulated degranulation and cytokine production is impaired in BMMC derived from *PI3KC2β*^*-/-*^ mice. A. 1 X 10^6^ PI3KC2β^+/+^ and PI3KC2β^-/-^ BMMCs were plated into 96 well plates, senstitized with anti-DNP IgE, and then stimulated with various concentrations of DNP-HSA for 30 minutes and β-hexosaminadase release into the supernatants are shown after correcting for spontaneous release is shown. ***p<0.001 or *p<0.05 as compared to the release measured in *PI3KC2β*^*+/+*^ and *PI3KC2β*^*-/-*^ at the same concentration. B. As described in (A) cells were stimulated with DNP-HSA for various times and induction of (i)a TNFα, (ii)a IL-6, and (iii)a IL-13 mRNA was assessed by RT-PCR. B. (i)b, (ii)b, (iii)b As described in (A), cells were stimulated for 24 hrs and supernatant was collected before and after stimulation by DNP-HSA. ELISA ready-set-go kit from ebioscience was used and induction of (i)b TNFα, (ii)b IL-6, and (iii)b IL-13 was assessed in the supernatant as per manufacturer’s protocol. ***p<0.001, **p<0.01 or *p<0.05 as compared to *PI3KC2β*^*+/+*^ and *PI3KC2β*^*-/-*^ at the same time point. All experiments shown are representative of at least three experiments performed on cells isolated from three separate mice. *p<0.05 as compared to the release measured in *PI3KC2β*^*+/+*^ at the same concentration.

Ca^2+^ influx plays a key role in mediating degranulation and cytokine production and release by mast cells[2, 3]. While basal β-hexosaminidase released between PI3KC2β^-/-^ and PI3KC2β^+/+^ BMMC was similar, it was significantly decreased in PI3KC2β^-/-^ BMMCs following FcεRI stimulation (Fig 2A). In addition, FcεRI stimulated induction of mRNA (Fig 2B (i)a-(iii)a) as well as protein (Fig 2B (i)b-(iii)b) for the cytokines TNFα, IL-6, and IL-13 respectively was also decreased in PI3KC2β^-/-^ BMMCs.

### *PI3KC2β*^*-/-*^ mice exhibit decreased susceptible to acute immediate anaphylaxis

**Fig 3:**
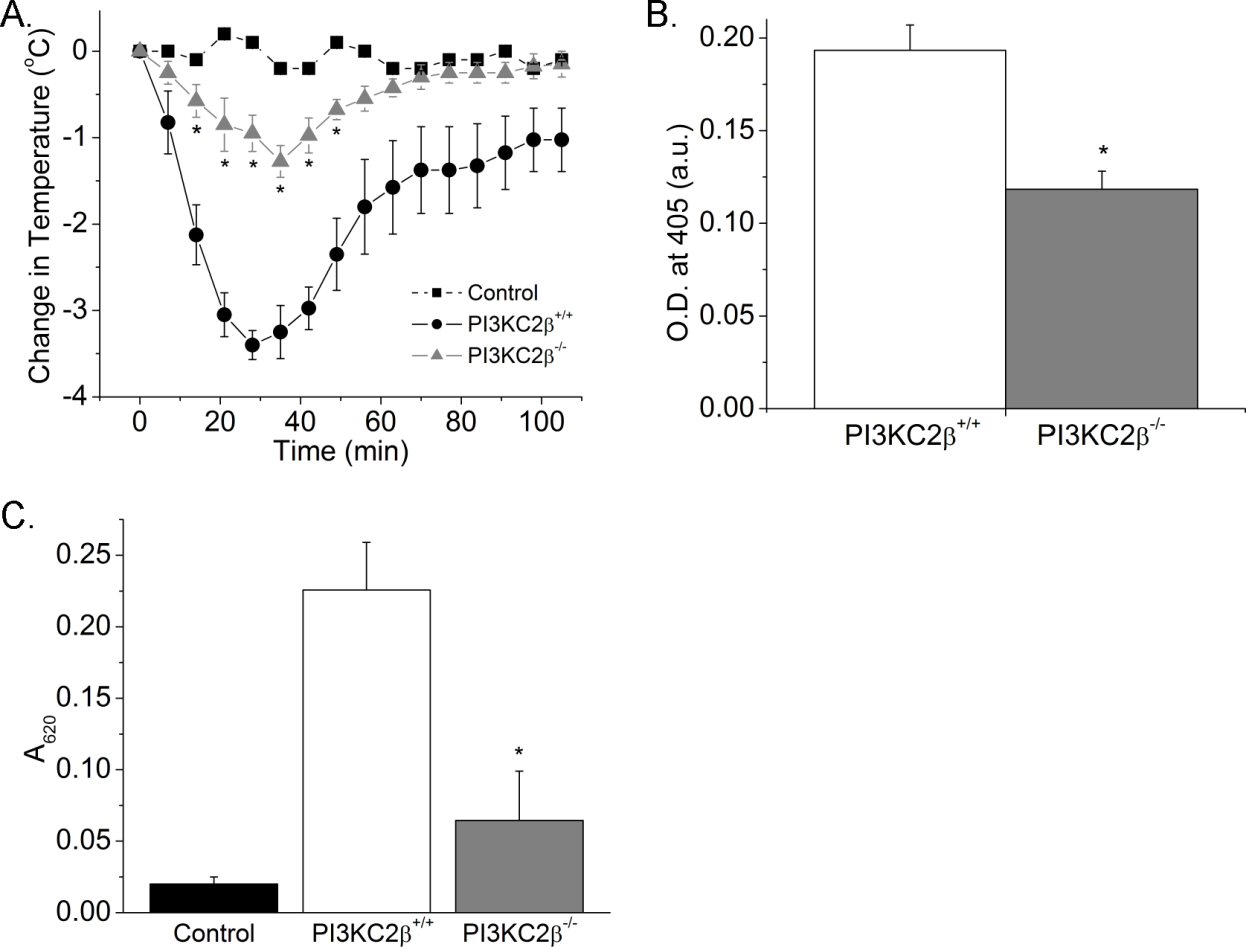
Passive and systemic anaphylaxis in *PI3KC2β*^*-/-*^ mice. A. Mean decrease in body temperature (^0^C) of *PI3KC2β*^*+/+*^ and *PI3KC2β*^*-/-*^ mice following induction of anaphylaxis (n = 5 mice in each group). B. Mean serum histamine levels 30 minutes after induction of anaphylaxis (n = 5 mice in each group). *p<0.05 as compared to the *PI3KC2β*^*+/+*^. C. Passive cutaneous anaphylaxis in *PI3KC2β*^*+/+*^ and *PI3KC2β*^*-/-*^ mice. Data are expressed as A_620_ per gram of skin (n = 5 mice in each group). ∗p<0.05 compared to results for *PI3KC2β*^*+/+*^ mice.

To assess whether changes in BMMCs *in vitro* is also relevant *in vivo*, *PI3KC2β*^*-/-*^ *and PI3KC2β*^*+/+*^ mice were sensitized intraperitoneally (IP) with anti-DNP IgE and subsequently challenged IP with DNP-HSA or saline control 5 hours later and body temperature and serum histamine levels were assessed as previously described[7]. The decrease in body temperature and histamine release at 30 minutes following treatment with antigen was significantly decreased in *PI3KC2β*^*-/-*^ mice in comparison to *PI3KC2β*^*+/+*^ mice indicating that PI3KC2β plays a critical role in passive systemic anaphylaxis (Fig 3A and 3B). *PI3KC2β*^*-/-*^ mice are also less sensitive to passive cutaneous anaphylaxis (Fig 3C). Thus, these findings provide strong support that inhibiting PI3KC2β in vivo may provide benefit and treating IgE mediated disease.

## Discussion

Despite the generation of knockout mice for all 3 of the class II PI3K, only a limited number of studies have assessed the *in vivo* roles for these kinases in the context of disease models. Based on previous findings that siRNA knockdown of PI3KC2β led to impaired FcεRI activation of BMMCs in vitro*[7, 24]*, we undertook experiments in *PI3KC2β*^*-/-*^ mice to assess the role of PI3KC2β in IgE mediated anaphylaxis *in vivo*. The importance in understanding signaling pathways that mediate mast cell activation *in vivo* is highlighted by the many studies demonstrating critical roles for mast cells in not only allergy and anaphylaxis but also in the regulation of innate and adaptive immune responses[2]. Thus, identification of new, safe, and potent pharmacologic inhibitors of mast cells will likely provide new therapies to treat a variety of different diseases. Studying *PI3KC2β*^*-/-*^ mice and BMMCs derived from these mice, we now show that activation of PI3KC2β plays a critical role for mast cell activation *in vitro* and *in vivo* in 2 mouse models of anaphylaxis.

Most of the previous studies on PI3K in mast cells have focused on the class I PI3Ks which are heterodimers consisting of a catalytic and a regulatory subunit. Of the class I PI3K, the catalytic subunits of the class 1A p110δ and the class 1B p110γ have been most closely associated with regulating mast cell degranulation, cytokine production and *in vivo* mast cell activation by FcεRI, Kit and G protein coupled receptors respectively[23, 25–27]. p110γ has been proposed to enhance activation of mast cells by functioning as an amplifier of G-protein coupled receptors such as adenosine[26] while p110δ is required for stem cell factor mediated proliferation and migration[23]. p110δ is also partially required for FcεRI stimulated release of β-hexosminadase and *in vivo* anaphylaxis although the pathways mediated by p110δ were not clearly defined[23]. However, the finding that these response were much more potently blocked by treatment with pan PI3 kinase inhibitors indicated that other PI3 kinase family members are also critical[23]. We now show that in addition to the class I PI3Ks, the class II PI3K PI3KC2β also plays a critical role in mediating mast cell degranulation and activation *in vivo*. However, in contrast to the class I PI3K that mediate activation through the generation of PI(3,4,5)P_3_ and is independent of Ca^2+^ influx, FcεRI activation of PI3KC2β leads to the generation of PI3P, which is then required for NDPK-B phosphorylation and activation of KCa3.1. This is supported by the finding that dialyzing PI3P into PI3KC2β^-/-^ BMMC is sufficient to rescue KCa3.1 current to levels seen in PI3KC2β^+/+^ BMMCs. Efflux of K^+^ via an active KCa3.1 facilitates extracellular Ca^2+^ influx via CRAC by maintaining a negative membrane potential, which is required to sustain a favorable electrical gradient for continued Ca^2+^ influx and subsequent mast cell activation[5].

In addition to a role for PI3KC2β, a recent study has demonstrated that PI3KC2α also plays a role in FcεRI stimulated degranulation of mast cells albeit by a different mechanism and different PI3 product[28]. While the exact mechanism whereby PI3KC2α mediates degranulation was not identified, PI3KC2α was required to generate PI(3,4)P_2_ on CD63 positive secretory granules, suggesting that generation of PI(3,4)P_2_ on the secretory vesicle is required for vesicle exocytosis. The generation of PI(3,4)P_2_ by PI3KC2α and PI3P by PI3KC2β following FcεRI activation is consistent with the ability of class II PI3Ks to generate both PI3P and PI(3,4)P_2_ under distinct conditions, but the extent and factors that determine which reaction is catalyzed by these enzymes are still largely unclear[12].

Our findings provide support that pharmacologically targeting PI3KC2β may be a novel therapy to treat a number of different diseases associated with activated mast cells. In addition, recent evidence that inhibition of PI3KC2β improves insulin sensitivity and glucose homeostasis[18], reverses the myopathy seen in mice mutant for myotubulin related protein 1 (MTM1)[29], and slows tumor growth[30] suggests that pharmacologic inhibitors of PI3KC2β may be beneficial in multiple different disease beyond their effects on mast cells. While PI3K inhibitors that only inhibit PI3KC2β have not yet been tested in animals, they will likely be extremely safe and well tolerated given that *PI3KC2β*^*-/-*^ mice[17] as well as mice that overexpress a kinase dead PI3KC2β[18] exhibit no overt phenotypes. In addition, previous studies have also demonstrated that PI3KC2β is also critical in T cell receptor activation of KCa3.1 and Ca^2+^ influx in subsets of CD4 T cells that include T helper 1 (Th1) and Th2 cells[24]. Thus, targeting PI3KC2β provides a means to target multiple different cell types that contribute to disease simultaneously with a single drug.
